# New crossroads for potential therapeutic intervention in cancer - intersections between CDCP1, EGFR family members and downstream signaling pathways

**DOI:** 10.18632/oncoscience.286

**Published:** 2016-01-29

**Authors:** Yaowu He, Brittney S. Harrington, John D. Hooper

**Affiliations:** ^1^ Cancer Biology and Management Program, Mater Research Institute–The University of Queensland, Translational Research Institute, Woolloongabba, Australia

**Keywords:** CDCP1, EGFR, HER2, Met, Src, antibody

## Abstract

Signaling pathways regulated by the receptor CDCP1 play central roles in promoting cancer and in mediating resistance to chemo- and targeted-therapies. In this perspective we briefly summarize these findings as well as data demonstrating poorer outcomes for several malignancies that exhibit elevated CDCP1 expression. Promising data from preclinical studies suggest that CDCP1 targeted agents, including therapeutic antibodies, could be useful in the treatment of cancer patients selected on the basis of activation of CDCP1 and its signaling partners including EGFR, HER2, Met and Src.

## INTRODUCTION

Over the last decade the membrane spanning glycoprotein CUB Domain Containing Protein 1 (CDCP1) has emerged as a potential therapeutic target for several cancers [1-3]. Consistent with roles in malignant progression its elevated expression correlates with poor outcome in malignancies of the kidney [4, 5], lung [6], colon and rectum [7], pancreas [8] and ovary [9]. Similarly there is accumulating evidence that CDCP1 is important in processes generic to cancer progression such as cell survival, migration and anchorage-independent growth, and that it is critical for dissemination of cancers that spread *via* vascular and transcoelomic routes [1-3]. Its potential as a target is supported by reports that five anti-CDCP1 antibodies, or antibody fragments, have demonstrated the ability, in *in vivo* models, to inhibit tumor growth or metastasis [10-13], with one of these antibodies successfully tested in a breast cancer tumor regression model [12].

**Figure 1:**
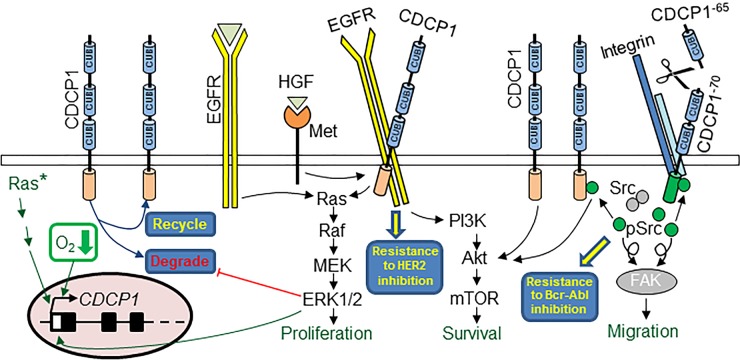
CDCP1-mediated and targetable pathways in cancer CDCP1 promotes cancer cell proliferation (generally under non-adherent conditions), survival and migration, and mediates resistance to chemo- and targeted-therapies. CDCP1 expression at the cell surface is increased by ligand activation of EGFR signaling, constitutively activated mutant Ras (Ras*), and hypoxia (O2↓). Scissors represent arginine/lysine specific serine proteases that cleave and activate CDCP1.

As highlighted in Figure 1, an emerging feature of CDCP1 biology is its intersection with pathways that are critical for cancer progression, and that dual targeting of CDCP1 and these other pathways could be beneficial against a range of malignancies. For example, EGF signalling, *via* EGFR to RAS/RAF/MEK/ERK, simultaneously stimulates CDCP1 expression and promotes CDCP1-mediated *in vitro* migration of ovarian cancer cells [14]. It has also been shown that EGF activation of EGFR further accentuates CDCP1-induced pro-cancer effects by inhibiting proteasome-mediated, palmitoylation-dependent constitutive degradation of CDCP1. This promotes recycling of CDCP1 to the cell surface, increasing its physical availability to mediate increased cell migration and, potentially, as a target for therapeutic antibodies [15]. CDCP1-mediated pro-cancer effects can also occur *via* ligand/receptor-independent activation of pathways downstream of cell surface EGFR family members. For example, mutations in the most frequently activated oncogenes, the *Ras* gene family, activate RAF/MEK/ERK which robustly induce CDCP1 mRNA and protein expression in non-small cell lung cancer (NSCLC) cells [16]. At least in NSCLC cells, p-Y-CDCP1 is required for Ras oncogenic functions including cell migration, invasion, colony formation in soft agar and resistance to anoikis *in vitro* [16]. Signaling *via* CDCP1, potentially *via* growth factor receptor axes, can be intensified under hypoxic conditions, a common microenvironment for cancer cells that also contributes to chemoresistance. In clear cell renal cell carcinoma (ccRCC) cells under hypoxic conditions, hypoxia-inducible factor (HIF)-2α induces the expression and activation of CDCP1, which relays pro-invasion and pro-tumor growth signals *via* PKCδ [5, 17]. These effects are potentially *via* the hypoxia-regulated and known HIF-2α targets EGFR and the HGF receptor, Met [5]. Induction of CDCP1 by HIF-2α under hypoxic conditions has also been demonstrated in hepatocellular carcinoma where elevated CDCP1 expression also correlates with poor patient survival [18].

Recent literature indicates that CDCP1 may also be a key mediator of resistance to therapies targeting EGFR family members. It has recently been shown that Met mediates resistance to the EGFR targeted therapies gefitinib and cetuximab [19]. HGF induced signalling efficiently confers this resistance in squamous cell carcinoma cells, with analysis of HGF-induced EGFR binding partners identifying direct interaction between EGFR and CDCP1 [19]. Although the role of EGFR/CDCP1 binding in resistance was not directly tested, interactions between these proteins were impervious to EGFR inhibition [19]. Consistent with a role in resistance to therapy, doxorubicin-induced apoptosis of prostate cancer cells *in vitro* is disrupted by CDCP1, with antibody blockade of CDCP1 restoring chemosensitivity [10]. Antibody targeting of CDCP1 can also markedly improve responsiveness to chemotherapy *in vivo*, as demonstrated for an intraperitoneal mouse xenograft of a clear cell ovarian carcinoma cell line treated with carboplatin [9]. Another powerful example of the role of CDCP1 in mediating poor response to therapy involves breast cancer resistance to the HER2 targeted therapeutic antibody trastuzumab. A quantitative proteomics screen comparing the phospho-tyrosine (p-Y) content of trastuzumab-sensitive and -resistant breast cancer cell lines identified 12 fold higher levels of p-Y-CDCP1 in resistant cells [20]. Confirming the role of CDCP1 in resistance in this model, siRNA mediated silencing of CDCP1 restored cell line responsiveness *in vitro* to trastuzumab [20]. Further supporting that CDCP1 is important in breast cancer resistance to trastuzumab, a more recent study demonstrated that CDCP1 and HER2 are co-overexpressed in 12% of primary and 30% of metastatic breast tumors, and that co-expression correlates with poorest disease free and overall survival [21]. Functionally, co-expression markedly increased orthotopic xenograft tumor growth in mice, and also drastically accentuated activation of both HER2 and HER3 *in vitro* as well as ligand-independent HER2 homodimerization, and heterodimerization of HER2/HER3 and HER2/EGFR [21]. Mechanistically the role of CDCP1 in recruiting Src to the plasma membrane appears to be a key contributor to trastuzumab resistance. CDCP1 binds directly to HER2 which is required for Src-mediated phosphorylation of HER2-Y877 and -Y1248 and EGFR-Y1068, as well as reciprocal HER2 phosphorylation of Src [21]. Importantly, both *in vitro* and *in vivo* the CDCP1 enhanced Src-HER2 interaction drove breast cancer trastuzumab resistance, with dual targeting of HER2 and Src able to overcome resistance in a mouse model [21]. The data provide a rationale for development of CDCP1-targeting agents that can be used in combination with anti-HER2 therapies for tumors double positive for HER2 and CDCP1 [21].

Interestingly, CDCP1-mediated activation of Src can be initiated by other distinct events. CDCP1 is synthesized as a 135 kDa transmembrane protein (CDCP1^−135^) that in cell lines *in vitro* and *in vivo*, and in human tumors, can be proteolytically cleaved producing a 70 kDa cell retained fragment (CDCP1^−70^), and two 65 kDa cell released fragments (CDCP1^−65^) [9, 22, 23]. While the roles of the CDCP1^−65^ fragments are unknown, both CDCP1^−70^ and CDCP1^−135^ interact with Src to transduce signals to a range of other proteins, generally *via* activation of PKCδ. In settings where cancer cells are required to survive without the support of a matrix, including growth in suspension, growth as spheroids or during dissemination, Src-dependent and -independent pathways activate PI3K/Akt signaling to promote cell survival and movement [8, 24–28]. For example, in a mouse intraperitoneal model of ovarian cancer CDCP1 pro-survival signalling to PI3K/Akt is Src independent [9], whereas in animal models of vascular metastasis, proteolytic cleavage of CDCP1 induces Src phosphorylation of CDCP1^−70^ which complexes with activated β1-integrin to signal *via* Src to FAK-PI3K-Akt to suppress PARP-1-induced apoptosis [10, 22, 29]. It is apparent that in these, and potentially other oncogenic settings, CDCP1 is the major substrate of Src family kinases (SFKs) and that it actively competes for Src with other mediators of pro-cancer phenotypes such as FAK [30]. There is evidence that interactions between CDCP1 and SFKs can also mediate resistance to therapy. A recent study demonstrated that CDCP1 is increased ∼9 fold in chronic myeloid leukemia cells that are resistant to the selective Bcr-Abl kinase inhibitor nilotinib, and that silencing of CDCP1 markedly improved responses to nilotinib [31].

In summary, CDCP1 has important and targetable roles in cancer. The growing literature demonstrating that CDCP1 expression is often elevated in cancer and that this overexpression correlates with poor outcome for various malignancies, sets the challenge for the development of effective CDCP1 targeted therapies. In particular, such agents have potential, in carefully selected patient cohorts, to improve responsiveness to therapies that target EGFR family members and Src family kinases, and to delay or even circumvent development of resistance to these drugs.
